# Retrospective study on the benefit of adjuvant radiotherapy in men with intraductal carcinoma of prostate

**DOI:** 10.1186/s13014-019-1267-3

**Published:** 2019-04-25

**Authors:** Vincent Q. Trinh, Nazim Benzerdjeb, Ségolène Chagnon-Monarque, Nicolas Dionne, Guila Delouya, André Kougioumoutzakis, Jennifer Sirois, Roula Albadine, Mathieu Latour, Anne-Marie Mes-Masson, Hélène Hovington, Alain Bergeron, Kevin C. Zorn, Yves Fradet, Fred Saad, Daniel Taussky, Dominique Trudel

**Affiliations:** 1 Department of Pathology, Centre hospitalier de l’Université de Montréal, 1051 Sanguinet, Montréal, Québec H2X 0C1 Canada; 2 Centre de recherche du Centre hospitalier de l’Université de Montréal and Institut du cancer de Montréal, 900 Saint-Denis, Montréal, Québec H2X 0A9 Canada; 3 Department of Radiation Oncology, Centre Hospitalier de l’Université de Montréal, 1051 Sanguinet, Montréal, Québec H2X 0C1 Canada; 4 Department of Medicine, Université de Montréal, Montréal, Québec H3C 3J7 Canada; 5 Laboratoire d’Uro-Oncologie Expérimentale, Centre de Recherche du Centre Hospitalier Universitaire de Québec-Université-Laval, Hôpital L’Hôtel-Dieu de Québec, 10 McMahon, Québec, G1R 3S1 Canada; 6 Department of Urology, Centre Hospitalier de l’Université de Montréal, 1051 Sanguinet, Montréal, Québec H2X 0C1 Canada; 7 Department of Urology, Centre Hospitalier Universitaire de Québec-Université-Laval, Hôpital L’Hôtel-Dieu de Québec, 11 Côte du Palais, Québec, G1R 2J6 Canada

**Keywords:** Adjuvant radiotherapy, Biochemical recurrence, Prostate cancer, Radical prostatectomy

## Abstract

**Background:**

Intraductal carcinoma of the prostate (IDC-P) is an independent biomarker of recurrence and survival with particular treatment response, yet no study has tested its response to radiotherapy. The aim of our project was to test the impact of adjuvant radiotherapy (ART) in patients with localized to locally advanced prostate cancer (PC) and IDC-P.

**Materials and methods:**

We performed a retrospective study of men with pT2-T3 PC treated by radical prostatectomy (RP) with or without ART, from two centres (1993–2015). Exclusion criteria were the use of another type of treatment prior to biochemical recurrence (BCR), and detectable prostate- specific antigen (PSA) following RP or ART. Primary outcome was BCR (2 consecutive PSA ≥ 0.2 ng/ml). Patients were grouped by treatment (RP_only_/RP + ART), IDC-P status, and presence of high-risk features (HRF: Grade Groups 4–5, positive margins, pT3 stage).

**Results:**

We reviewed 293 RP specimens (median follow-up 99 months, 69 BCR). Forty-eight patients (16.4%) were treated by RP + ART. Multivariate Cox regression for BCR indicated that IDC-P had the strongest impact (hazard ratio [HR] = 2.39, 95% confidence interval [CI]:1.44–3.97), while ART reduced the risk of BCR (HR = 0.38, 95%CI: 0.17–0.85). Other HRF were all significant except for pT3b stage. IDC-P[+] patients who did not receive ART had the worst BCR-free survival (log-rank *P* = 0.023). Furthermore, IDC-P had the same impact on BCR-free survival as ≥1 HRF (log-rank *P* = 0.955).

**Conclusion:**

Men with IDC-P who did not receive ART had the highest BCR rates, and IDC-P had the same impact as ≥1 HRF, which are often used as ART indications. Once validated, ART should be considered in patients with IDC-P.

**Electronic supplementary material:**

The online version of this article (10.1186/s13014-019-1267-3) contains supplementary material, which is available to authorized users.

## Introduction

Intraductal carcinoma of the prostate (IDC-P) is a histologic subtype detected in up to 20% of routine radical prostatectomies (RPs) [1, 2]. IDC-P is mostly found alongside high-grade high volume and/or advanced acinar adenocarcinoma, and is independently associated with poor prognosis and shorter biochemical recurrence (BCR)-free survival [3–6]. Notably, IDC-P incurs a 20-month reduction in overall survival in metastatic castration-resistant prostate cancer (PC) [7]. IDC-P also incurs a 29% reduced disease-specific survival at 15 years in patients with a Gleason score (GS) ≥7 [8]. Due to the clinical relevance of IDC-P, it is now mandatory to indicate its presence regardless of grade [9].

Recent treatment response studies of patient-derived xenografts have demonstrated that IDC-P can withstand androgen deprivation with an associated rapid emergence of castrate-tolerant cells [10]. It also does not respond to treatment in the same fashion as usual PC without IDC-P. In low-grade organ-confined PC, IDC-P has been associated with a high risk of progression [11]. Yet, to our knowledge, no treatment response studies exist for IDC-P in non-metastatic PC, even though it often progresses towards early distant metastases and reduced survival [12].

Adjuvant radiotherapy (ART) is an option for high-risk localized and locally advanced PC [13, 14]. Its advantages mainly outweigh prominent side effects in PC that are prone to high recurrence rates [15]. Currently, guidelines suggest that ART should be considered for PC exhibiting high-risk features (HRF): positive margins (PMs), extraprostatic extension (EPE), seminal vesicle invasion (SVI), and sometimes Grade Groups (GG) 4 & 5 [16]. However, IDC-P to date has not been factored into the therapeutic decision-making process for patients at high risk of recurrence [15, 17–19]. We therefore explored whether patients with IDC-P respond to ART and compared its impact against currently acknowledged HRF.

## Materials and methods

### Cohorts and clinical data

RP specimens were collected from the PC biorepository of the Centre de recherche du Centre hospitalier de l’Université de Montréal (Centre 1), and the PC biorepository of the Laboratoire d’Uro-Oncologie Expérimentale of the Centre de recherche du Centre hospitalier universitaire Québec-Université Laval (Centre 2). Inclusion criteria were the following: initial diagnosis from 1993 until 2015, men with localized to locally advanced PC (pT2–3, pN0-NX, cN0M0), men treated by first-line RP with ART (RP + ART) from both centres, and men treated by first-line RP only (RP_only_, without ART) from Centre 2. Only patients treated by RP with ART were extracted from center 1, as that database contains more than 4000 RP specimens and it would be unfeasible to review all patients treated by RP only. Exclusion criteria were the following: use of neoadjuvant therapy, use of adjuvant androgen-deprivation therapy or adjuvant chemotherapy, detectable prostate-specific antigen (PSA) following RP, and patients who had missing data for any of these criteria. Recorded clinical data included age at diagnosis, date of diagnosis, PSA progression, imaging data, and delay prior to treatments. Ethics board approval was obtained prior to biobanking and data extraction for the present study.

### Histopathological review of radical prostatectomy specimens

**Fig. 1:**
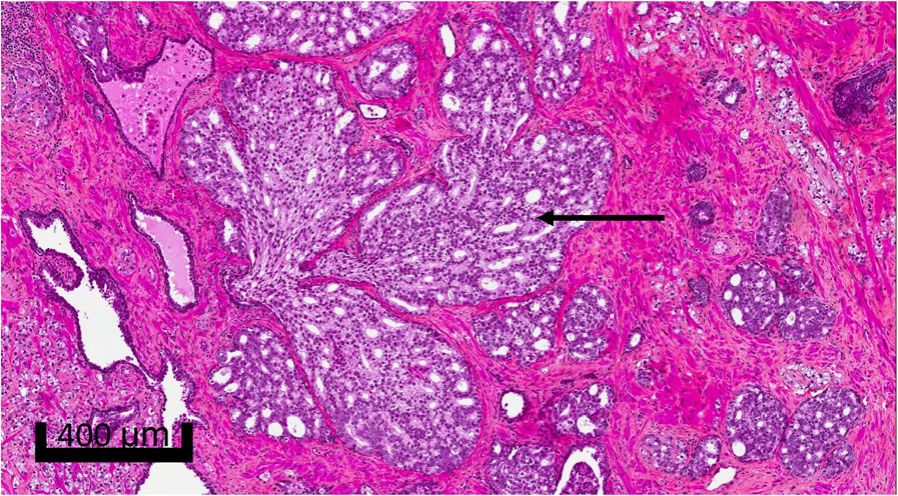
Routine hematoxylin and eosin (200X) of intraductal carcinoma of the prostate. Shown is the defining dense cribriform proliferation in a dilated duct/acinus with preservation of basal cells (arrow)

Since IDC-P is often a focal finding, at least 75% of slides from formalin-fixed paraffin-embedded (FFPE) blocks had to be available for analysis [20]. All slides were independently reviewed by two pathologists without knowledge of clinical information. They assessed tumour grading, pathological staging, and IDC-P identification. The modified Gleason grading system/Grade Group (GG) grading was used to grade the invasive component, and pathological staging was performed according to the seventh edition of the American Joint Committee on Cancer’s Prostate Staging System [21, 22]. IDC-P was diagnosed according to Guo and Epstein’s criteria, which includes an intraductal proliferation of solid or dense cribriform patterns; or loose cribriform with markedly enlarged nuclei, marked pleomorphism, frequent mitotic figures and frequent comedonecrosis (Fig. 1) [3].

Immunohistochemistry was performed if paraffin-embedded blocks were available and when the intraductal nature of the lesion was uncertain or found with mimicking architecture such as large acinar cribriform patterns. Unwaxed 4-μm sections were subjected to heat-mediated epitope retrieval, followed by incubation with a cocktail of antibodies directed against p63/34βE12/p504s and counterstaining with hematoxylin (BenchMark Ultra, Ventana Medical Systems, United States).

Recorded histological data included GG, lymphovascular invasion (LVI), EPE, SVI, PM, and the presence of IDC-P. The GG reclassifies GS according to significant prognostic groups: GG 1 = GS 6; GG 2 = GS 3 + 4; GG 3 = GS 4 + 3; GG 4 = GS 8; GG 5 = GS 9 & 10 [9].

### Adjuvant radiation therapy protocols

The same ART approach was used in both academic centres. A dose of 66 Gy in 2-Gy fractions was administered to the tumour bed according to the Radiation Therapy Oncology Group (RTOG) guidelines [23]. Margins around the clinical targeting volume were 7–10 mm. Inclusion of pelvic lymph nodes in the treatment volume was left to the discretion of the treating physician.

### Patient grouping

Patients were grouped according to the presence of HRF (GG 4–5, EPE, SVI and PM), which were shown to benefit the most from ART [14].

### Outcome

The main endpoint was BCR during follow-up, broadly defined as two consecutive PSA test results over 0.2 ng/ml. As per exclusion criteria, serum PSA levels had to be undetectable after RP.

### Statistical analysis

Clinical and pathological data were tested with univariate and multivariate analyses with SPSS v23.0. Univariate methods included Fisher’s exact test, Pearson’s Chi-square test, Welch’s T-test, and Mann-Whitney U test. Survival analyses were tested with the Kaplan-Meier method, log-rank test, and Cox regression analysis. Basic SPSS code was used in the analyses.

## Results

### Clinical and histopathological baseline characteristics

**Fig. 2:**
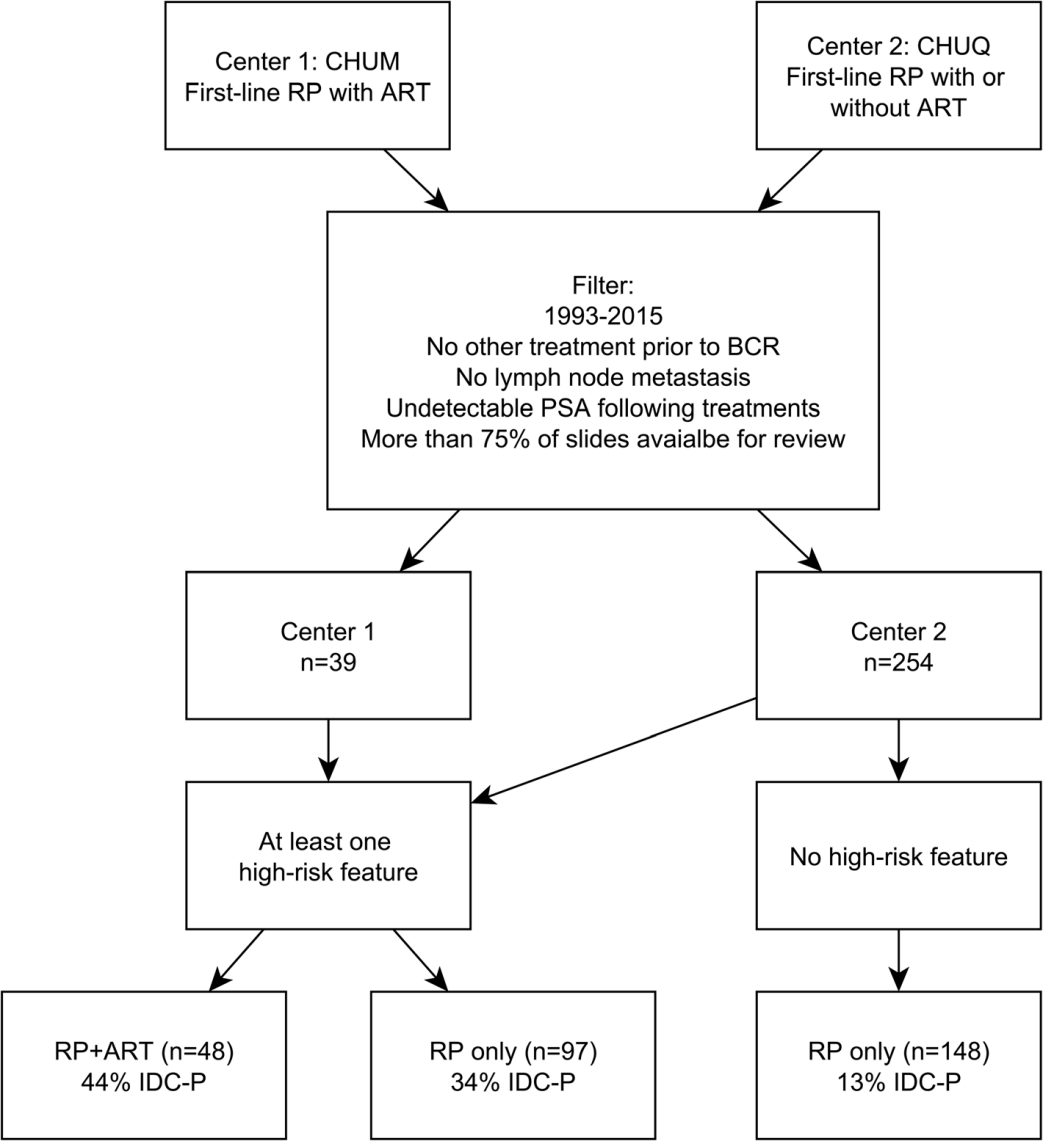
Study workflow. For the main study purpose, patients were mainly separated according to the presence of high-risk features (Grade groups 4–5, seminal vesicle invasion, extraprostatic extension, positive margins). Abbreviations: CHUM: Centre hospitalier de l’Université de Montréal (Centre 1); CHUQ: Centre hospitalier universitaire de Québec-Université Laval (Centre 2); RP: radical prostatectomy; IDC-P: intraductal carcinoma of prostate

The general workflow of the study is illustrated in Fig. 2. There were 73 patients with IDC-P (Table 1). IDC-P was consistently associated with higher odds of HRF. In order to compare treatment effect in survival curves, patients were then stratified by HRF, IDC-P status, and treatment group (Table 2). There were 148 patients without any HRF, while 145 patients had ≥1 HRF. IDC-P was present in 12.8% (19/129) of patients without any HRF. In patients with ≥1 HRF, 43.8% (21/54) of RP + ART patients had IDC-P, while 34.4% (27/91) of RP_only_ patients had IDC-P. RP + ART patients had shorter follow-up times since ART was offered more frequently in recent years at both our institutions. Among patients with ≥1 HRF, the RP + ART group had higher GG as well as higher incidences of LVI, EPE and SVI. Table 1 describes the baseline characteristics sub-stratified according to IDC-P status. IDC-P[+] groups were consistently associated with a higher rate of HRF when compared to their IDC-P[−] counterparts.

**Table 1:** Baseline characteristics of patients according to presence of IDC-P

	Patients with IDC-P (*n* = 73)	Patients without IDC-P (*n* = 220)	*P* ^*^
Adjuvant radiotherapy	21 (29%)	27 (12%)	0.002
Mean age at diagnosis (S.D.)	62.1 (6.8)	62.0 (6.8)	0.852
Grade group			
1	16 (22%)	113 (51%)	< 0.001
2	26 (36%)	82 (37%)	
3	21 (29%)	19 (9%)	
4	2 (3%)	1 (1%)	
5	8 (11%)	5 (2%)	
Lymphovascular invasion	19 (26%)	17 (8%)	< 0.001
Extraprostatic extension	42 (58%)	66 (30%)	< 0.001
Seminal vesicle invasion	15 (21%)	14 (6%)	0.001
Positive margins	29 (40%)	51 (23%)	0.010
Mean # of high-risk features (S.D.)	1.32 (1.1)	0.6 (0.9)	< 0.001
Median follow-up (months) (I.Q.R.)	61 (29–101)	112 (70–140)	< 0.001
Centre			
1	19 (26%)	20 (9%)	< 0.001
2	54 (74%)	200 (91%)	

High-risk features: Grade groups 4–5, seminal vesicle invasion, positive margins, extraprostatic extension

*S.D* Standard deviation, *I.Q.R* Interquartile range

^*^Welch’s test for means, Fisher’s exact test for 2 × 2 tests, Pearson’s chi-square for categorical variables with more than 2 categories, Mann-Whitney U for medians

**Table 2:** Comparative baseline characteristics of patients, stratified according to presence of high-risk features, treatments and IDC-P status

	At least one high risk feature (*n* = 145)					No high-risk feature (*n* = 148)		
	RP + ART, IDC-P[+] (*n* = 21)	RP + ART, IDC-P[−] (*n* = 27)	RP only, IDC-P[+] (*n* = 33)	RP only, IDC-P[−] (*n* = 64)	*P* ^*^	RP only, IDC-P[+] (*n* = 19)	RP only, IDC-P[−] (*n* = 129)	*P* ^*^
Mean age at diagnosis (S.D.)	60.9 (5.8)	62.1 (5.7)	62.3 (6.5)	61.7 (6.0)	0.848	63.3 (8.1)	62.1 (7.4)	0.554
Grade Group								
1	0 (0%)	2 (7%)	8 (24%)	26 (41%)	< 0.001	8 (42%)	85 (66%)	0.134
2	7 (33%)	15 (56%)	10 (30%)	31 (48%)		9 (47%)	36 (28%)	
3	8 (38%)	7 (26%)	11 (33%)	4 (6%)		2 (11%)	8 (6%)	
4	1 (5%)	0 (0%)	1 (3%)	1 (2%)		0	0	
5	5 (24%)	3 (11%)	3 (9%)	2 (3%)		0	0	
Lymphovascular invasion	12 (57%)	9 (33%)	6 (18%)	7 (11%)	< 0.001	1 (5%)	1 (1%)	0.241
Extraprostatic extension	20 (95%)	23 (85%)	22 (67%)	43 (67%)	0.026	0	0	–
Seminal vesicle invasion	9 (43%)	7 (26%)	6 (18%)	7 (11%)	0.013	0	0	–
Positive margins	12 (57%)	16 (59%)	17 (52%)	35 (56%)	0.942	0	0	–
Mean #of high-risk features (S.D.)	2.2 (1.0)	1.8 (0.8)	1.5 (0.7)	1.4 (0.6)	< 0.001	0	0	–
Median follow-up (months) (I.Q.R.)	49 (30–67)	44 (23–65)	61 (24–123)	108 (56–153)	< 0.001	89 (61–116)	118 (84–139)	0.685
Centre								
1	19 (91%)	20 (74%)	0	0	< 0.001	0	0	–
2	2 (9%)	7 (26%)	33 (100%)	64 (100%)		19	129	

High-risk features: Grade groups 4–5, seminal vesicle invasion, positive margins, extraprostatic extension

*ART* Adjuvant radiotherapy, *IDC-P* Intraductal carcinoma of the prostate, *RP* Radical prostatectomy, *S.D.* Standard deviation, *I.Q.R.* Interquartile range

^*^Welch’s test for means, Fisher’s exact test for 2 × 2 tests, Pearson’s chi-square for categorical variables with more than 2 categories, Mann-Whitney U test and Kruskall-Wallis for medians

### Cox regression analysis of all patients

Of the total 293 patients (median follow-up time: 99 months, interquartile range [IQR]: 53–136), 69 men experienced BCR. Results from a Cox regression analysis of BCR including ART, GG, IDC-P, EPE, SVI, and PM are presented in Table 3. All factors except for SVI demonstrated a significant effect. IDC-P was observed to have the strongest negative effect for BCR risk in our model (hazard ratio [HR] = 2.39, 95% confidence interval [CI]: 1.44–3.97, *P* = 0.001). ART had a protective effect with an HR = 0.38 (95% CI: 0.17–0.85, *P* = 0.018). We then performed these analyses comparing patients with IDC-P separately from patients without IDC-P (Additional file 1: Table S1). General trends persist, but the effect of ART trends towards a stronger effect in patients with IDC-P compared to patients without IDC-P. Indeed, in patients with IDC-P, ART seems to have the most significant absolute effect on outcome, while margin status has the strongest effect in patients without IDC-P. Of note, we found no evidence that IDC-P or ART affected BCR outcomes differently between strata based on the other factors (ART/IDC-P respectively, and GG, EPE, PM, SVI) (Additional file 1: Table S2).

**Table 3:** Cox regression analysis for the prediction of biochemical recurrence in all patients from two academic centres

	All patients (*n* = 293, 69 BCR)	
	Hazard ratio (95% CI)	*P*
Intraductal carcinoma of prostate	2.39 (1.44–3.97)	0.001
Adjuvant radiation therapy	0.38 (0.17–0.85)	0.018
Grade group	1.28 (1.00–1.62)	0.046
Extraprostatic extension	1.84 (1.07–3.15)	0.027
Positive margins	2.12 (1.28–3.50)	0.003
Seminal vesicle invasion	1.35 (0.67–2.69)	0.400

*IDC-P* Intraductal carcinoma of the prostate, *CI* Confidence interval

### BCR-free survival in patients with ≥1 HRF

**Fig. 3:**
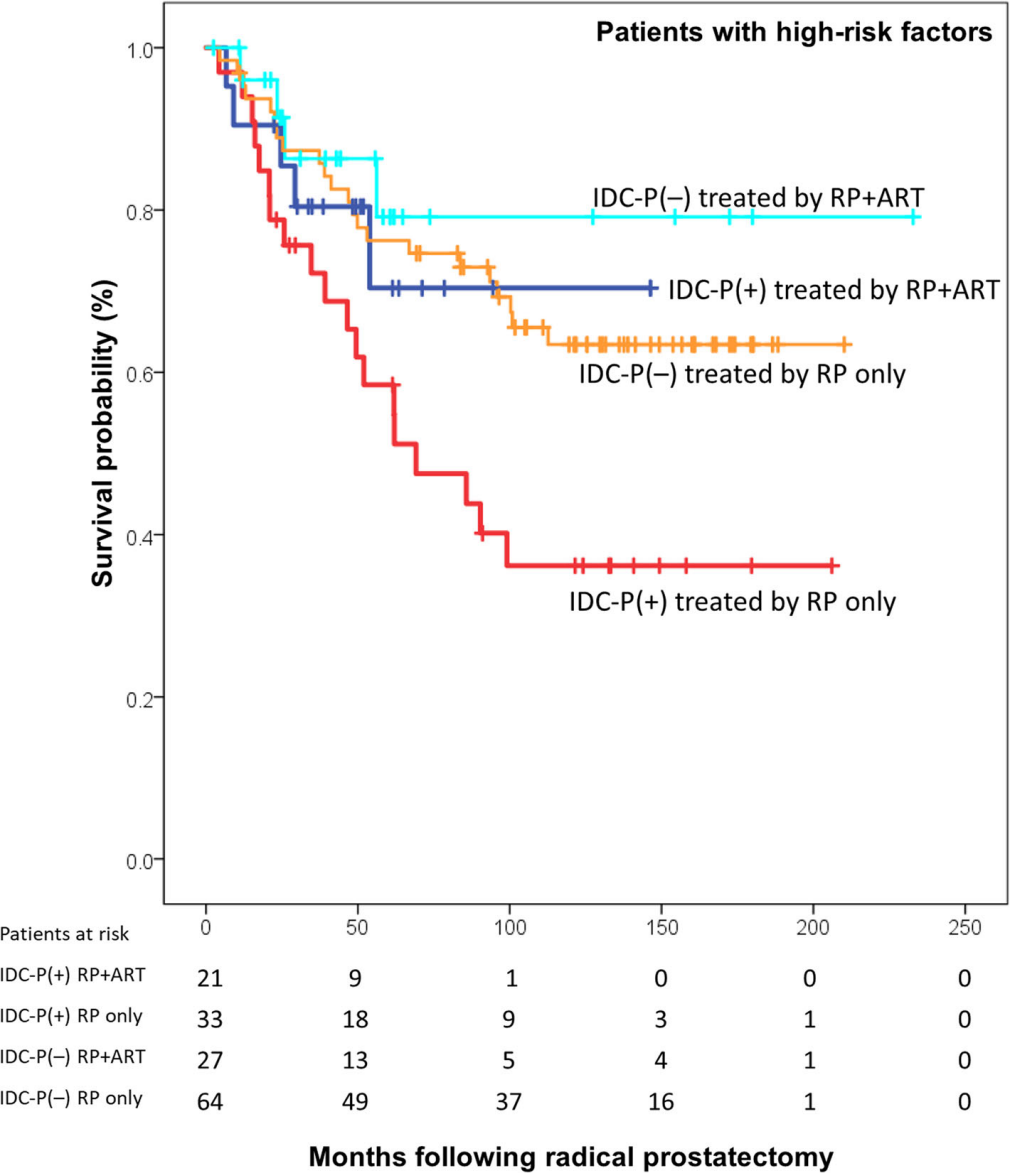
Kaplan-Meier curve of BCR-free survival following radical prostatectomy in patients with at least one high-risk feature, according to IDC-P and treatment status. Patients treated by RP_only_ and who had IDC-P were the outlying group with poor outcome. Log-rank testing *P* = 0.023. When comparing IDC-P patients only (red and blue curves), mean survival of RP + ART patients was 112 months (95% CI: 85–138, median not yet reach), and median survival of RP_only_ patients was 69 months (95% CI: 28–111), with log-rank testing *P* = 0.158. Abbreviations: IDC-P: intraductal carcinoma of prostate. RP: radical prostatectomy. ART: adjuvant radiotherapy. HRF: High-risk features (GG4–5, seminal vesicle invasion, positive margins, extraprostatic extension)

In this subset of patients with HRF, sub-groups were based on IDC-P status and treatments in order to have comparative analysis groups. A BCR-free survival Kaplan-Meier curve with log-rank testing is presented in Fig. 3 for men with ≥1 HRF. The median follow-up time in these patients was 67 months (IQR: 31–131). Patients with IDC-P who did not receive ART had the poorest outcome, as 64% experienced BCR at 10 years, while only 29.6% of patients with IDC-P who received ART experience BCR at 10 years. However, log-rank testing did not reach significance (*P* = 0.158) in this small subgroup analysis (*n* = 54). Noteworthy, IDC-P[+] patients treated by RP_only_ fared worst, yet they significantly had lower rates of EPE (*P* = 0.018) and LVI (*P =* 0.007) compared to IDC-P[+] patients treated by RP + ART (Table 2). They did however have similar rates of positive margins (IDC-P[+] treated by RP + ART: 57%, IDC-P[+] treated by RP only: 52%).

### Impact of IDC-P in patients without any HRF: IDC-P as a potential stand-alone criterion for ART

**Fig. 4:**
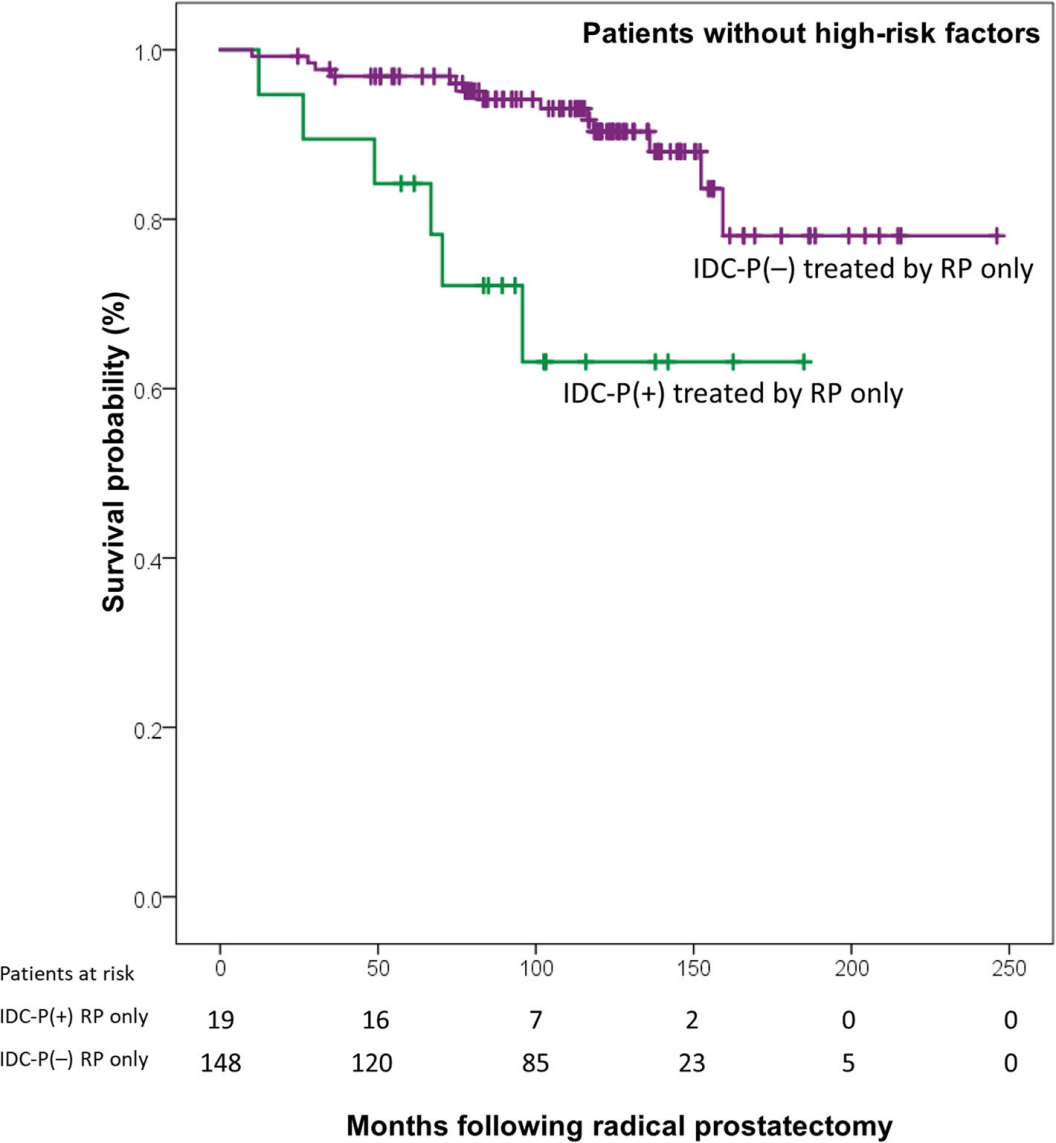
Kaplan-Meier curve of BCR-free survival following radical prostatectomy of patients without any high-risk features. Mean survival of IDC-P[+] patients was 138 months (95% CI:108–169, median survival not yet reached), and mean survival of IDC-P[−] patients was 218 months (95% CI:202–234, median survival not yet reached). Log-rank testing *P* < 0.002. Abbreviations: IDC-P: intraductal carcinoma of the prostate. RP: radical prostatectomy. ART: adjuvant radiotherapy. High-risk features: Grade groups 4–5, seminal vesicle invasion, positive margins, extraprostatic extension

Since ART can be offered to patients with PM, pT3 disease, and high-grade localized PC [14], we tested IDC-P’s stand-alone impact on BCR in patients without those HRF, who would have never been considered for ART (*n* = 148, 19 BCR, median follow-up time of 116 months [IQR: 83–139]). The baseline characteristics of these patients are summarized in Table 2. Figure 4 shows the Kaplan-Meier curve of patients without any HRF, which demonstrate a difference in BCR-free survival. IDC-P[+] patients have a higher risk of BCR (IDC-P[+] = 37% versus IDC-P[−] = 10% at 10 years, log-rank *P* = 0.002). We further performed a Cox regression analysis to compare the role of IDC-P and of GG in men without HRF for the prediction of BCR. IDC-P was significantly associated with BCR (HR = 3.24, 95%CI: 1.20–8.77, *P* = 0.021), while GG was near significance (HR = 1.94, 95%CI: 0.99–3.79, *P* = 0.052).

**Fig. 5:**
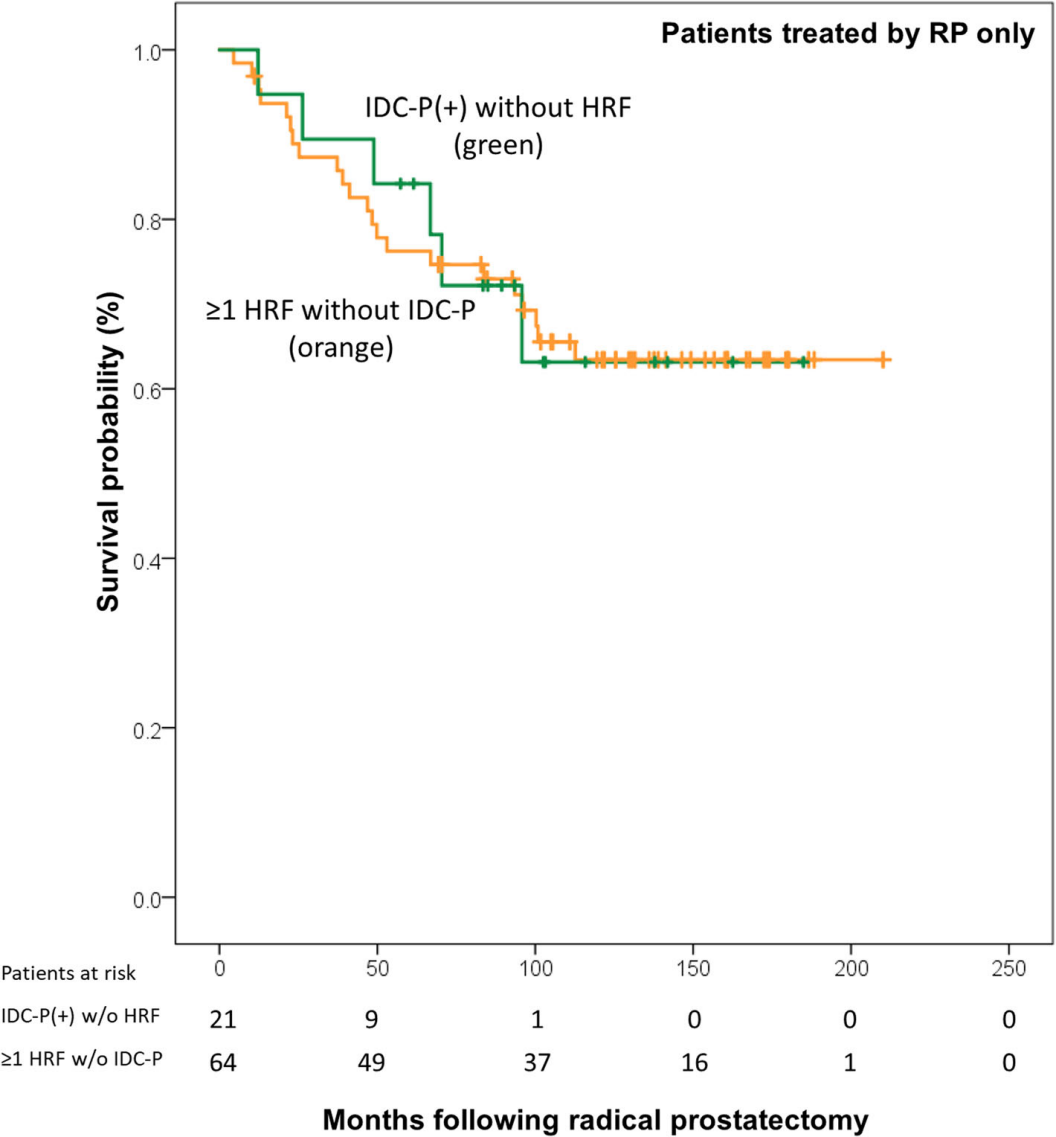
Kaplan-Meier curve of BCR-free survival following radical prostatectomy, comparing the effect of IDC-P (without any high-risk features) against patients with at least one high-risk feature (without IDC-P). Mean survival of ≥1 HRF patients was 152 months (95% CI: 133–172, median survival not yet reached), and mean survival of IDC-P[+] patients was 138 months (95% CI: 107–169, median survival not yet reached). Log-rank testing *P* = 0.955. Abbreviations: IDC-P: intraductal carcinoma of prostate. RP: radical prostatectomy. ART: adjuvant radiotherapy. HRF: High-risk features (Grade groups 4–5, seminal vesicle invasion, positive margins, extraprostatic extension)

We then tested if IDC-P had the same impact as ≥1 HRF [14]. In patients treated by RP_only_, a first group was composed of patients with IDC-P without any HRF, and the second group was composed of patients with ≥1 HRF without IDC-P. Both groups of patients had the almost the same rate of BCR at 10 years (36.8% for IDC-P[+] and 36.6% for ≥1 HRF, log-rank *P* = 0.955, Fig. 5).

## Discussion

IDC-P is a prevalent finding that negatively and independently affects recurrence-free and disease-specific survivals. However, there is a paucity of data about treatment response for non-metastatic PC with IDC-P [7, 10]. In parallel, ART has been observed to improve survival in patients with PC and is a viable option for aggressive and locally advanced disease [24]. In this study, we therefore suggest that against the currently recognized HRFs, for which ART is often considered, IDC-P confers a strong negative impact on BCR risk, an impact which is comparable to the effect of ≥1 HRF, while ART appears to portend a strong protective effect.

Our results are concordant with multiple studies that show a beneficial effect for ART and that also show the detrimental effect of IDC-P on survival, although they were analyzed separately [15, 17–20, 25, 26]. Additionally, IDC-P’s impact on BCR risk was estimated to be of 2.39 by Murata et al., as well as of 2.98 by Trudel et al. in a different cohort, on par with our results [20, 27].

Interestingly, as IDC-P showed the strongest deleterious effect on BCR compared to other HRFs, patients with this finding might be the most prone to benefit from ART. Actually, many other recent studies also show that IDC-P has a stronger effect on clinical progression than any other factor, including Gleason grade 5 and SVI [28–30]. These results highlight the need for the validation of the effect of aggressive treatment, notably, radiation therapy, in the presence of IDC-P.

Altogether, these results complement current literature concerning patient selection for ART. This process is focused on balancing ART’s reduction of recurrence rates, with its significant side effects [14]. Current literature suggests that patients with residual cancer at margins seem to benefit the most from ART [14]. In addition, it can also be considered in men with SVI, EPE and GG4–5, as ART seem to improve their recurrence rates [14, 31]. However, our results suggest that the relative importance of these factors is confounded by IDC-P status. This is particularly true as these clinicopathological factors are sometimes inadequate to guide treatment in seemingly low-grade PC which progresses unexpectedly. Additional factors are needed to distinguish those vulnerable men from truly indolent cancer. IDC-P is an excellent candidate, as it is readily available in any standard laboratory, easy to detect by pathologists, and present in 13% of our patients with seemingly low-grade GG1 and GG2 PC lacking HRF. Altogether, we strongly believe that IDC-P deserves a more prominent role in treatment algorithms.

Since PC can be treated using very different and evolving modalities and can experience a wide spectrum of outcomes, we attempted to mitigate the intrinsic limitations of this retrospective study. We limited our study to a specific set of patients treated by RP (with or without ART) while excluding all men who received other treatments before BCR. Even though RP techniques have varied from 1993 until 2015, progression-related outcomes have not varied between RP approaches [32]. Similarly, the technical aspects of ART have remained largely unchanged between centres and throughout time [33]. Similarly, patients selected for pelvic lymphatic pathway irradiation during ART were not identified in the databases. However, it is worth noting that treatment approaches from both centers lead to similar outcomes [34]. Also, since PSA increases following RP can be unpredictable and prone to many confounders, we limited our cohort to patients who had undetectable PSA following RP. This approach would isolate the effect of postoperative ART. Another limitation was that the number of patients studied in each centre was unbalanced, as it was not feasible to review more than an estimated 1600 eligible patients only treated by RP from center 1. This is why we only extracted patients treated by ART from this much larger cohort. Patients from center 2 were not selected, and recruited prospectively. Moreover, the overall study group, though unbalance, showed comparable results in prevalence of IDC-P and other PC pathological factors. The follow-up time for patients with ART was also shorter, as this treatment was used more extensively in recent years in both centers. However, the second set of analyses of IDC-P as a stand-alone indicator for ART showed consistent results, and did not rely on this subgroup of patients. Importantly, we used BCR as an endpoint, despite the fact that the time to PC specific death after BCR is known to be variable [35]. As our study was aimed to explore whether patients with IDC-P respond to ART and to compare the impact of IDC-P against currently recognized HRFs, the use of BCR allowed us to perform those comparisons in a small study sample and to avoid the effect of treatments subsequent to BCR, but overall definitely calls for validation in larger cohorts.

## Conclusions

Our data has demonstrated higher biochemical failure in men with IDC-P who were not treated by ART. Additionally, IDC-P conferred the same level of BCR risk as current HRF of PC, which are often used as indicators for ART. If confirmed in other larger cohorts, current treatment algorithms should be modified to include IDC-P as a key factor leading to ART.

## Additional file

Additional file 1: **Table S1.** Sub-group stratified analysis comparing the risk of biochemical recurrence according to the presence of IDC-P. **Table S2.** Interaction analysis. (DOCX 22 kb)

## Abbreviations

ART: Adjuvant radiotherapy
BCR: Biochemical recurrence
CI: Confidence interval
EPE: Extraprostatic extension
FFPE: Formalin-fixed paraffin-embedded
GG: Grade Groups
GS: Gleason score
HR: Hazard ratio
HRF: High-risk features
IDC-P: Intraductal carcinoma of the prostate
LVI: Lymphovascular invasion
PC: Prostate cancer
PM: Positive margins
PSA: Prostate- specific antigen
RP: Radical prostatectomy
SVI: Seminal vesicle invasion
