# Supplementary material for: Retrospective study on the benefit of adjuvant radiotherapy in men with intraductal carcinoma of prostate
Source: Radiat Oncol. 2019 Apr 25;14:60. doi: 10.1186/s13014-019-1267-3 (PMC6482557; doi:10.1186/s13014-019-1267-3)
Supplement: Supplementary file 1 — Table S1. Sub-group stratified analysis comparing the risk of biochemical recurrence according to the presence of IDC-P. Table S2. Interaction analysis. (DOCX 22 kb) [file 13014_2019_1267_MOESM1_ESM.docx]

Additional file 1

**Table S1.** Sub-group stratified analysis comparing the risk of biochemical recurrence according to the presence of IDC-P.

|  | **Patients with IDC-P**  **(n=73, 30 BCR)** | | **Patients without IDC-P**  **(n=220, 39 BCR)** | |
| --- | --- | --- | --- | --- |
|  | **Hazard ratio (95% CI)** | ***P*** | **Hazard ratio (95% CI)** | ***P*** |
| Adjuvant radiation therapy | 0.31 (0.10-0.95) | 0.040 | 0.56 (0.18-1.77) | 0.324 |
| Grade groups | 1.37 (0.97-1.93) | 0.076 | 1.14 (0.80-1.62) | 0.471 |
| Extraprostatic extension | 1.53 (0.69-3.41) | 0.300 | 1.99 (0.94-4.19) | 0.072 |
| Positive margins | 1.71 (0.79-3.70) | 0.172 | 2.48 (1.26-4.89) | 0.008 |
| Seminal vesicle invasion | 1.16 (0.46-2.91) | 0.748 | 1.69 (0.58-4.92) | 0.337 |

IDC-P: Intraductal carcinoma of the prostate

BCR: Biochemical recurrence

CI: Confidence interval.

**Table S2.** Interaction analysis.

The Cox regression with 6 predictors was performed. All second order interactions with IDC-P and ART predictors were tested at the 0.15 level. We report the hazard ratios and 95% CI for each factor interacted with IDC-P or ART regardless of whether the interactions were statistically significant.

| **Interaction added to the main model** | ***P*** | **Contrast** | **Hazard ratio  (95% CI)** | ***P*** |
| --- | --- | --- | --- | --- |
| IDC-P*ART | 0.35 | ART 1 vs 0 if IDC-P=0 | 0.57 (0.19-1.73) | 0.32 |
|  |  | ART 1 vs 0 if IDC-P=1 | 0.29 (0.10-0.81) | 0.02 |
|  |  | IDC-P 1 vs 0 if ART=0 | 2.61 (1.53-4.45) | 0.0004 |
|  |  | IDC-P 1 vs 0 if ART=1 | 1.33 (0.35-4.98) | 0.68 |
| IDC-P*GG | 0.90 | GG (1-unit increase) if IDC-P=0 | 1.26 (0.91-1.74) | 0.16 |
|  |  | GG (1-unit increase) if IDC-P=1 | 1.30 (0.94-1.77) | 0.11 |
|  |  | IDC-P 1 vs 0 if GG=1 | 2.25 (0.77-6.53) | 0.14 |
|  |  | IDC-P 1 vs 0 if GG=2 | 2.31 (1.12-4.78) | 0.02 |
| IDC-P*EPE | 0.49 | EPE 1 vs 0 if IDC-P=0 | 2.13 (1.08-4.22) | 0.03 |
|  |  | EPE 1 vs 0 if IDC-P=1 | 1.51 (0.70-3.27) | 0.30 |
|  |  | IDC-P 1 vs 0 if EPE=0 | 2.90 (1.38-6.06) | 0.005 |
|  |  | IDC-P 1 vs 0 if EPE=1 | 2.05 (1.05-4.00) | 0.04 |
| IDC-P*PM | 0.34 | PM 1 vs 0 if IDC-P=0 | 2.62 (1.35-5.06) | 0.004 |
|  |  | PM 1 vs 0 if IDC-P=1 | 1.62 (0.76-3.43) | 0.21 |
|  |  | IDC-P 1 vs 0 if PM=0 | 2.96 (1.52-5.76) | 0.001 |
|  |  | IDC-P 1 vs 0 if PM=1 | 1.83 (0.86-3.88) | 0.16 |
| IDC-P*SVI | 0.51 | SVI 1 vs 0 if IDC-P=0 | 1.75 (0.64-4.77) | 0.28 |
|  |  | SVI 1 vs 0 if IDC-P=1 | 1.14 (0.47-2.73) | 0.78 |
|  |  | IDC-P 1 vs 0 if SVI=0 | 2.58 (1.48-4.49) | 0.0008 |
|  |  | IDC-P 1 vs 0 if SVI=1 | 1.68 (0.53-5.34) | 0.38 |
| ART*GG | 0.60 | GG (1-unit increase) if ART=0 | 1.31 (1.01-1.70) | 0.04 |
|  |  | GG (1-unit increase) if ART=1 | 1.13 (0.68-1.88) | 0.64 |
|  |  | ART 1 vs 0 if GG=1 | 0.61 (0.09-4.10) | 0.61 |
|  |  | ART 1 vs 0 if GG=2 | 0.53 (0.13-2.16) | 0.37 |
| ART*EPE | 0.75 | EPE 1 vs 0 if ART=0 | 1.87 (1.08-3.24) | 0.02 |
|  |  | EPE 1 vs 0 if ART=1 | 1.31 (0.16-11.01) | 0.81 |
|  |  | ART 1 vs 0 if EPE=0 | 0.52 (0.07-4.01) | 0.53 |
|  |  | ART 1 vs 0 if EPE=1 | 0.37 (0.16-0.86) | 0.02 |
| ART*PM | 0.69 | PM 1 vs 0 if ART=0 | 2.20 (1.29-3.77) | 0.004 |
|  |  | PM 1 vs 0 if ART=1 | 1.61 (0.39-6.60) | 0.51 |
|  |  | ART 1 vs 0 if PM=0 | 0.47 (0.13-1.62) | 0.23 |
|  |  | ART 1 vs 0 if PM=1 | 0.34 (0.13-0.91) | 0.03 |
| ART*SVI | 0.46 | SVI 1 vs 0 if ART=0 | 1.15 (0.50-2.65) | 0.74 |
|  |  | SVI 1 vs 0 if ART=1 | 2.07 (0.54-7.91) | 0.29 |
|  |  | ART 1 vs 0 if SVI=0 | 0.30 (0.10-0.88) | 0.03 |
|  |  | ART 1 vs 0 if SVI=1 | 0.54 (0.16-1.79) | 0.31 |

ART: adjuvant radiotherapy.

CI: Confidence interval.

IDC-P: intraductal carcinoma of the prostate.

EPE : extra-prostatic extension

GG : grade groups

PM: positive margins

SVI: seminal vesicle invasion
